# Association Between Serum α-Klotho Levels and Habitual Physical Activity in Hemodialysis Patients: A Pilot Clinical Study

**DOI:** 10.3390/jcm15114341

**Published:** 2026-06-04

**Authors:** Misa Miura, Osamu Ito, Shigeru Oowada, Nobuyuki Endo, Masahiro Kohzuki, Teruhiko Maeba

**Affiliations:** 1 Faculty of Health Sciences, Tsukuba University of Technology, Tsukuba 305-8521, Japan; 2 Division of General Medicine and Rehabilitation, Faculty of Medicine, Tohoku Medical and Pharmaceutical University, Sendai 981-8558, Japan; oito@tohoku-mpu.ac.jp; 3 Asao Clinic, Asao-ku, Kawasaki 215-0004, Japan; shigegigioowada0324@nifty.com (S.O.); tmaeba@tkh.att.ne.jp (T.M.); 4 Research and Development Department, The Wakasa Wan Energy Research Center, Tsuruga 914-0135, Japan; nendou@werc.or.jp; 5 Yamagata Prefectural University of Health Sciences, Yamagata 990-2212, Japan; makohzuki@gmail.com

**Keywords:** serum α-Klotho levels, hemodialysis, exercise therapy, high-intensity interval training, chronic kidney disease, anti-aging, renal rehabilitation

## Abstract

**Background/Objectives:** Chronic kidney disease (CKD) is characterized by accelerated aging and functional decline. Serum α-Klotho levels, an anti-aging biomarker predominantly associated with renal function, have emerged as potential indicators of biological aging and cardiovascular risk. To investigate the association between serum α-Klotho levels and habitual physical activity in hemodialysis patients. **Methods:** This study combined (1) a prospective case analysis of high-intensity interval training (HIIT) in a hemodialysis patient and (2) a cross-sectional analysis of 24 hemodialysis patients and 18 healthy controls. Serum α-Klotho levels were measured using ELISA, and their association with habitual physical activity was evaluated. **Results:** Serum α-Klotho levels were significantly lower in hemodialysis patients than in healthy controls (*p* < 0.001). In hemodialysis patients, physical activity was moderately correlated with serum α-Klotho levels (r = 0.52, *p* = 0.02), whereas no significant association was observed in healthy controls. The case analysis demonstrated marked improvement in physical function following HIIT. These findings suggest that serum α-Klotho levels may be associated with physical activity status in hemodialysis patients. **Conclusions:** Serum α-Klotho levels were associated with habitual physical activity in hemodialysis patients and may represent a potential molecular indicator related to physical function and rehabilitation status. These findings support further investigation of biomarker-informed approaches in renal rehabilitation.

## 1. Introduction

Chronic kidney disease (CKD) is increasingly recognized as a state of accelerated aging, characterized by sarcopenia, vascular dysfunction, and increased mortality. In Japan, CKD affects approximately one in seven to eight adults [1], and the number of elderly patients undergoing dialysis continues to rise according to the annual statistical survey by the Japanese Society for Dialysis Therapy [2].

Understanding the relationship between physical activity and Serum α-Klotho levels may provide a novel framework for integrating molecular biomarkers into renal rehabilitation [3,4]. Beyond functional outcomes, recent attention has focused on the molecular mechanisms underlying these benefits [5].

α-Klotho is a well-established anti-aging protein predominantly expressed in the distal renal tubules [6]. It regulates phosphate metabolism, oxidative stress, and endothelial function [7]. Reduced serum α-Klotho levels are associated with CKD progression, cardiovascular disease, and mortality [8].

Emerging evidence suggests that exercise may be associated with increased circulating serum α-Klotho levels [9]. However, clinical data in hemodialysis patients remain scarce [10].

Therefore, this study aimed to explore the association between habitual physical activity and serum α-Klotho levels in hemodialysis patients using a combined case study and cross-sectional approach.

## 2. Methods

### 2.1. Study Design

This study consisted of two components:

(1) a prospective case study evaluating the effects of high-intensity interval training (HIIT), and (2) a cross-sectional study investigating the association between exercise habits and serum α-Klotho levels.

### 2.2. Participants

Case Study: The case study involved a male patient (Table 1) in his 60s undergoing maintenance hemodialysis for more than 10 years. For the case study, the patient was selected as a representative case from a consecutive cohort of patients who initiated the HIIT protocol, based on his clinical requirement for functional recovery. The primary renal disease was IgA nephropathy. The patient had stable hemodynamic status and was deemed eligible for rehabilitation by the attending physician. The patient was receiving standard medications, including antihypertensive agents. No changes in regular medications were made before or after the intervention period.

Cross-Sectional Study: The cross-sectional study included 24 patients undergoing maintenance hemodialysis and 18 healthy controls (Table 2). The biochemical parameters of the study participants are shown in Table 2. Participants were recruited using a consecutive sampling method. All hemodialysis patients who visited the clinic during the study period and met the inclusion criteria were invited to participate to minimize selection bias.

### 2.3. Exercise Intervention (Case Study)

The HIIT program consisted of resistance and aerobic training, following established protocols for renal rehabilitation [11]. Resistance training was performed using power rehabilitation machines. Resistance training was performed using power rehabilitation machines targeting knee flexion/extension and hip abduction/adduction. The protocol included:

Low-intensity exercise: 10 repetitions × 2 sets at 11 on the Borg Rating of Perceived Exertion (RPE) scale.

High-intensity exercise: 10 repetitions × 1 set at 14–15 RPE [12].

Aerobic exercise was conducted using a cycle ergometer [11]:

Low-intensity intervals: 2 min × 4 sets at 11–13 RPE.

Moderate-to-high intensity intervals: 1 min × 2 sets at 13–14 RPE.

In addition, a 5 min whole-body stretching routine was performed as part of both the warm-up and cool-down phases.

### 2.4. Measurement of Serum α-Klotho Levels

Serum α-Klotho levels were measured using a commercially available enzyme-linked immunosorbent assay (ELISA) kit (Immuno-Biological Laboratories Co., Ltd., Fujioka, Japan), according to the manufacturer’s instructions. This assay has been previously validated for its precision and reliability in clinical populations [13,14]. Plasma samples were processed and analyzed following the standardized protocol [15], and values were expressed in pg/mL.

### 2.5. Assessment of Exercise Habits

Exercise habits were assessed through structured interviews. Participants were asked about the type, duration, and frequency of their usual physical activities. Exercise intensity was quantified using the Compendium of Physical Activities [16], and MET values were calculated accordingly to estimate physical activity levels [17,18].

### 2.6. Statistical Analysis

Statistical analyses were performed using IBM SPSS Statistics (version 27.0; IBM Corp., Armonk, NY, USA) [19]. Group comparisons were performed using the Mann–Whitney U test. Associations between exercise habits and serum α-Klotho levels were analyzed using Spearman’s rank correlation coefficient. The area under the curve (AUC) was calculated to assess the predictive power of the model [20]. A *p*-value < 0.05 was considered statistically significant.

### 2.7. Sample Size Calculation

This study was designed as an exploratory pilot study. The sample size was determined based on feasibility and prior similar studies investigating serum α-Klotho levels and exercise interventions in CKD populations [21,22]. In the cross-sectional analysis, a sample size of approximately 20 participants per group is considered sufficient to detect moderate to large correlations (r ≥ 0.5) with a statistical power of 0.8 and a significance level of 0.05 [23]. Therefore, the present sample size (hemodialysis: *n* = 24, controls: *n* = 18) was considered appropriate for detecting clinically meaningful associations. Although the sample size was determined based on previous studies and a power of 0.8, it remains relatively small for a cross-sectional analysis. Therefore, this study is positioned as an exploratory pilot study, and while the results provide meaningful preliminary insights, the generalizability of the findings should be interpreted with caution.

### 2.8. Rationale for Study Design

A combined case study and cross-sectional design was adopted to provide both mechanistic and clinical insights [24]. The case study allowed detailed evaluation of the effects of a structured HIIT intervention on functional outcomes in a real-world clinical setting [25]. The cross-sectional component enabled the investigation of associations between habitual physical activity and serum α-Klotho levels in a broader population. This combined design was chosen to bridge individual-level intervention effects and population-level associations, thereby providing a translational perspective in renal rehabilitation [26].

## 3. Results

**Figure 1:**
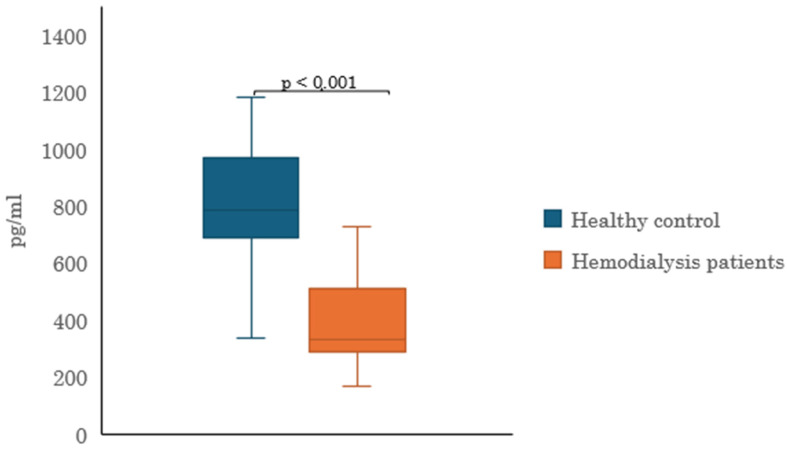
Comparison of serum α-Klotho levels between healthy controls and hemodialysis patients. Serum α-Klotho levels (pg/mL) were significantly lower in hemodialysis patients (*n* = 24) compared to healthy controls (*n* = 18). Data are presented as box plots showing the median, quartiles, and range. This finding is consistent with previous reports showing reduced α-Klotho levels in dialysis populations. The analysis confirmed low intra-assay variability, supporting the reliability of the ELISA measurements. A moderate positive correlation was observed between exercise habits and serum α-Klotho levels in hemodialysis patients (r = 0.52, *p* = 0.02), whereas no significant association was found in healthy individuals (Figure 2).

In the case study, the HIIT intervention led to a measured functional recovery. The HIIT program was completed without any adverse events, such as intradialytic hypotension or excessive fatigue. The patient’s Barthel Index improved from 90 to 100, and the SPPB score increased from 10 to 12. Peak exercise intensity rose from 2.0 to 5.5 METs (Table 1), and the patient resumed agricultural work. In the cross-sectional analysis, serum α-Klotho levels were significantly lower in hemodialysis patients than in healthy controls (*p* < 0.001) (Figure 1).

**Figure 2:**
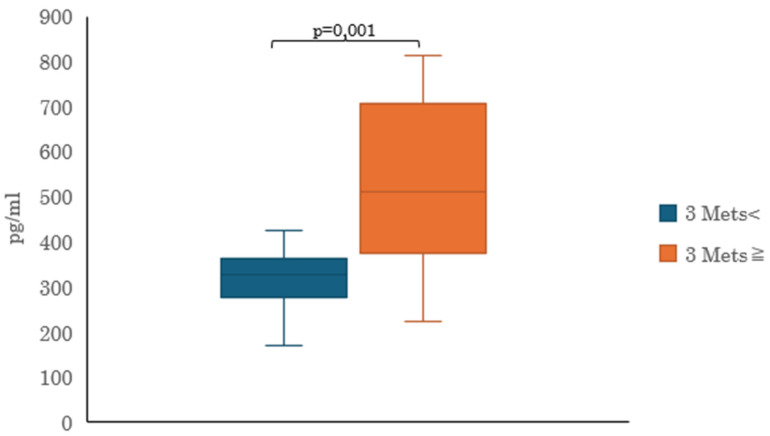
Impact of habitual physical activity on serum α-Klotho levels in hemodialysis patients. Hemodialysis patients with habitual physical activity (≥3 METs, *n* = 11) exhibited significantly higher serum α-Klotho levels compared to those with low physical activity (<3 METs, *n* = 13). *p* = 0.001 by the Mann–Whitney U test. These findings suggest that serum α-Klotho levels may reflect differences in habitual physical activity in patients undergoing maintenance hemodialysis. Receiver operating characteristic (ROC) analysis in hemodialysis patients using a cutoff value of 450 pg/mL for serum α-Klotho levels demonstrated high sensitivity (1.00) but low specificity (0.125) for identifying individuals with habitual physical activity (≥3 METs). The Youden index was 0.125, indicating limited discriminative ability of this cutoff value. These findings show that although serum α-Klotho levels are associated with exercise habits in hemodialysis patients, a single cutoff value may not be sufficient for accurate clinical classification.

## 4. Discussion

This study provides preliminary insights into the association between habitual physical activity and serum α-Klotho levels in hemodialysis patients. To our knowledge, this is one of the first clinical studies linking serum α-Klotho levels with exercise behavior in hemodialysis patients. The case study demonstrated that HIIT was associated with substantial improvement in physical function, even in patients with long-term dialysis [27].While the patient’s physical function had previously declined during periods of self-monitored home exercise, the structured HIIT intervention was associated with marked functional improvement and return to agricultural work [28]. Specifically, the patient’s Barthel Index (BI) score improved from 90 to 100, and the SPPB score increased from 10/12 to a full mark of 12/12 [26]. Importantly, the cross-sectional findings revealed a significant association between exercise habits and serum α-Klotho levels, specifically in hemodialysis patients [29]. Although the age differences and diabetes prevalence between groups are recognized as study limitations, our ROC analysis suggested that while serum α-Klotho levels are associated with physical activity, a single cutoff value might have limited practical utility for clinical screening due to low specificity. Nevertheless, the significant correlation (r = 0.52) and the clinical recovery observed in the case study suggest that serum α-Klotho levels remain a potentially relevant molecular indicator associated with habitual physical activity. [30,31]. The observed association between habitual physical activity and serum α-Klotho levels may reflect differences in physiological reserve and functional status among hemodialysis patients [32]. From a translational perspective, this study links macro-level functional recovery and micro-level molecular observations [33]. These findings highlight the potential clinical relevance of exercise therapy, particularly HIIT, in renal rehabilitation.

This study has several limitations. First, there was a significant age difference between the hemodialysis group and the healthy control group, which may have influenced the baseline serum α-Klotho levels. Although age differences may influence baseline serum α-Klotho levels, the observed association within the hemodialysis group suggests a potential association between physical activity and serum α-Klotho levels. Despite this mismatch, this exploratory pilot study provides preliminary evidence regarding the association between physical activity and serum α-Klotho levels. Second, the ROC analysis revealed that while serum α-Klotho levels were associated with exercise habits, the specificity was notably low (0.125). This suggests that serum α-Klotho levels alone may have limited practical utility as a standalone clinical screening tool for physical activity. Parameters related to mineral metabolism and vascular calcification, including calcium, phosphate, and parathyroid hormone levels, were not systematically evaluated in this exploratory study. Future studies incorporating these variables are warranted. Nevertheless, the moderate correlation (r = 0.52) and the dramatic functional recovery observed in our case study suggest that serum α-Klotho levels remain a meaningful molecular indicator that is associated with habitual physical activity and functional status.

## 5. Conclusions

Higher habitual physical activity was associated with higher serum α-Klotho levels in hemodialysis patients. These findings support further investigation of molecular biomarkers in renal rehabilitation.

## Figures and Tables

**Table 1:** Clinical Progress and Outcomes of the Case Study Patient.

Variable	Pre-Intervention	Post-Intervention (HIIT)
Barthel Index (BI)	90/100	100/100
SPPB score	10/12	12/12 (Full score)
Max Exercise Intensity	2.0 METs	5.5 METs
MMT (Lower extremity)	4	4~5
Lower back pain (NRS)	5~6/10	0/10 (Disappeared)
Blood Pressure	Stable	Stable (No change)
serum α-Klotho levels (pg/mL)	342.2	517.9

**Table 2:** Baseline Characteristics of Participants in the Cross-sectional Study.

Variable	Hemodialysis (*n* = 24)	Healthy Controls (*n* = 18)	*p*-Value
Age (years)	75.5 ± 6.8	50.7 ± 11.4	0.001
Male (%)	25%	16.70%	0.64
Prevalence of diabetes	40%	0	N/A
Primary disease	Diabetic Nephropathy IgA Nephropathy Nephrosclerosis Polycystic kidney disease Idiopathic	-	N/A
serum α-Klotho levels (pg/mL)	446.6 ± 174.6	858.8 ± 361.2	<0.001
Whole PTH (pg/mL)	87.6 ± 52.6	N/A	-
Ca (mg/dL)	9.0 ± 0.5	N/A	-
P (mg/dL)	5.0 ± 1.1	N/A	-

## Data Availability

All data generated or analyzed during this study are included in this published article.
